# Neither random nor censored: estimating intensity-dependent probabilities for missing values in label-free proteomics

**DOI:** 10.1093/bioinformatics/btad200

**Published:** 2023-04-17

**Authors:** Mengbo Li, Gordon K Smyth

**Affiliations:** Bioinformatics Division, The Walter and Eliza Hall Institute of Medical Research, Parkville, Victoria 3052, Australia; Department of Medical Biology, The University of Melbourne, Parkville, Victoria 3010, Australia; Bioinformatics Division, The Walter and Eliza Hall Institute of Medical Research, Parkville, Victoria 3052, Australia; School of Mathematics and Statistics, The University of Melbourne, Parkville, Victoria 3010, Australia

## Abstract

**Motivation:**

Mass spectrometry proteomics is a powerful tool in biomedical research but its usefulness is limited by the frequent occurrence of missing values in peptides that cannot be reliably quantified (detected) for particular samples. Many analysis strategies have been proposed for missing values where the discussion often focuses on distinguishing whether values are missing completely at random (MCAR), missing at random (MAR) or missing not at random (MNAR).

**Results:**

Statistical models and algorithms are proposed for estimating the detection probabilities and for evaluating how much statistical information can or cannot be recovered from the missing value pattern. The probability that an intensity is detected is shown to be accurately modeled as a logit-linear function of the underlying intensity, showing that missing value process is intermediate between MAR and censoring. The detection probability asymptotes to 100% for high intensities, showing that missing values unrelated to intensity are rare. The rule applies globally to each dataset and is appropriate for both high and lowly expressed peptides. A probability model is developed that allows the distribution of unobserved intensities to be inferred from the observed values. The detection probability model is incorporated into a likelihood-based approach for assessing differential expression and successfully recovers statistical power compared to omitting the missing values from the analysis. In contrast, imputation methods are shown to perform poorly, either reducing statistical power or increasing the false discovery rate to unacceptable levels.

**Availability and implementation:**

Data and code to reproduce the results shown in this article are available from https://mengbo-li.github.io/protDP/.

## 1 Introduction

Shotgun proteomics is a suite of bottom-up proteomics methods by which proteins in complex biological samples are identified and quantified using liquid chromatography coupled with tandem mass spectrometry (LC-MS/MS) (Liu et al. 2004; Zhang et al. 2013). Label-free quantification has gained popularity over label-based approaches as it involves less experimental preparation and has the capacity to process large numbers of samples (Cox et al. 2014; Navarro et al. 2016). In a typical workflow, protein contents extracted from samples are first broken down into peptide mixtures through proteolytic digestion. Peptides are then separated by liquid chromatography and analyzed by a mass spectrometer (Altelaar et al. 2013). Peptide precursors are identified by correlating fragment ion spectra produced in the tandem mass spectrometry (MS/MS) step to the theoretical MS/MS spectra predicted in a protein sequence database or a spectral library (Nesvizhskii et al. 2007). Peptide abundances are measured by MS intensities derived from peak heights or areas. Protein quantification is then achieved by summarizing intensities of constituent peptides. Despite advances in many aspects of MS-based proteomics, both instrumental and methodological, missing values in the peptide intensity quantifications remain a common feature of such datasets and present one of most challenging problems for downstream analysis (Webb-Robertson et al. 2015; Kong et al. 2022).

For proteomics data, the most likely missing value mechanisms are either (i) the expression level of the peptide precursor is below the detection limit of the instrument or (ii) the elution profile or spectral signature of the precursor cannot be distinguished from the signatures of other precursors in the same sample (Demichev et al. 2020). The second mechanism is highly stochastic because it depends on the extent to which other precursors with interfering profiles are expressed in the same sample. Both mechanisms are intensity-dependent because the precursor’s signature is more likely to be identified if the peptide is more highly expressed relative to similar peptides in the same sample. The subtlety of proteomics data is that there is no clear-cut or fixed detection limit and even very highly expressed peptides can be subject to missing values, so it is difficult to assign specific causes to particular observations. The missing value process therefore needs to summarized probabilistically in order to be modeled accurately.

It is a common observation in proteomics publications that the frequency of missing values tends to decrease with peptide abundance (Liu et al. 2004; Karpievitch et al. 2009; Webb-Robertson et al. 2015; Lazar et al. 2016; Liu and Dongre 2021). Luo et al. (2009) assumed the detection probability to be linearly dependent on its log-intensity on the logit scale. However, the proposed model has a complex hierarchical structure and is only suitable for iTRAQ data. O’Brien et al. (2018) assumed the detection probability to be probit-linear in the underlying log-intensity as part of a fully Bayesian differential expression approach.

In this article, we propose statistical models and algorithms for estimating the detection probabilities and for evaluating how much statistical information can or cannot be recovered from the missing value pattern. The detection probability curve (DPC) relates the probability of a peptide being detected (non-missing) to its underlying intensity, whether or not that intensity was observed. Using a wide variety of public datasets, we show that the DPC is very well approximated by a logit-linear relationship and that the proportion of values missing completely at random is very small. Using the DPC, we show that the unobserved intensity values follow a distribution with a shifted mean relative to the observed values, thus quantifying the bias that occurs when missing values are ignored. We derive the marginal probability of detection for each peptide in each condition and use this relationship to quantify the statistical information that can theoretically be recovered from the pattern of missing values in designed experiments. We show that the detection probability model can be incorporated into a likelihood-based approach for assessing differential expression and that this approach successfully recovers statistical power compared to omitting the missing values from the analysis. In contrast, imputation methods are shown to perform poorly, either reducing statistical power or increasing the false discovery rate to unacceptable levels.

## 2 Materials and methods

### 2.1 Datasets

#### 2.1.1 Dataset A: hybrid proteome data

Hybrid proteome samples were generated by mixing human, yeast and *Escherichia coli* lysates in different ratios and analyzed by SWATH-MS. For each mixture, triplicates were measured (Navarro et al. 2016). The original data were published accompanying the LFQbench software (Navarro et al. 2016) where details on sample preparation and data acquisition are available. We downloaded the HYE110 dataset (TripleTOF 6600; 64-variable-window acquisition) processed by DIA-NN under the library-based mode, with settings of DIA-NN detailed in Demichev et al. (2020). Precursor-level intensities were extracted from the DIA-NN report. For our analysis, we only consider the triplicates of Sample A (n=3). In this sub-sampled dataset, 34 689 precursor ions are detected in at least one sample, and the overall proportion of missing values is ∼8.6%. We also set intensity values <1 to be missing; this affected only a small number of values and had little impact on the percentage of missing values or on the results presented here. The log-2 transformation was applied to the intensities.

#### 2.1.2 Dataset B: cell cycle proteomes

Single-cell proteomes were profiled by the true single-cell-derived proteomics (T-SCP) pipeline as described in Brunner et al. (2022). Four cell populations enriched in different cell cycle stages were produced from HeLa cells by drug treatment (Brunner et al. 2022). Precursor ions in prepared samples were fragmented in the parallel accumulation–serial fragmentation with data-independent acquisition (diaPASEF) mode (Meier et al. 2020). The MS raw files were analyzed by DIA-NN in library-based mode (Demichev et al. 2020). Details on sample preparation, LC-MS/MS analysis and data processing are provided in Brunner et al. (2022). Processed data were downloaded from the ProteomeXchange Consortium via the PRIDE (Perez-Riverol et al. 2022) partner repository with the dataset identifier PXD024043. Precursor-level output was obtained from the DIA-NN report and cells from the cell cycle experiment were extracted (n=231). The number of detected precursor species is 10 754. About 60.4% of the data are missing values. We also set intensity values of zero to be missing; this affected only a small number of values and had little impact on the percentage of missing values. A log2-transformation was applied to the intensities.

#### 2.1.3 Dataset C: HepG2 technical replicate data

Technical replicates of HepG2 cell lysates were analyzed in Sinitcyn et al. (2021). Briefly, MS data were collected by data-independent acquisition (DIA) and analyzed by MaxDIA in discovery mode, a DIA data analysis software within MaxQuant (Cox and Mann 2008). Descriptions on sample preparation, LC-MS/MS procedures and the data processing workflow are described in Sinitcyn et al. (2021). Processed data were downloaded from the ProteomeXchange Consortium via the PRIDE (Perez-Riverol et al. 2022) partner repository with the dataset identifier PXD022589. Peptide-level data were obtained from the MaxQuant output, which reports missing values as zeros. Intensity values were log2-transformed. The number of detected precursors is 62 515 in 27 samples. The overall missing proportion is 32.8%.

#### 2.1.4 Dataset D: human blood plasma proteome

Human blood plasma samples from acute inflammation patients were collected and analyzed by Prianichnikov et al. (2020). In brief, MS data were acquired by data-dependent acquisition using the timsTOF Pro mass spectrometer operated on the PASEF scan mode. Raw MS data were then analyzed by MaxQuant. Details on sample preparation, LC-MS/MS procedures and the MaxQuant workflow are reported in Prianichnikov et al. (2020). Processed data are available from the ProteomeXchange Consortium via the PRIDE (Perez-Riverol et al. 2022) partner repository with the dataset identifier PXD014777. Peptide-level data were extracted from the MaxQuant output. Peptide species that had zero intensities in all samples were discarded. Intensity values were log2-transformed. The number of precursor species after filtering is 2384 in 212 samples. The overall missingness proportion is 56.9%.

### 2.2 Regression splines

Regression splines were fitted to the proportion of detected (non-missing) values for each precursor based on its average observed intensity. For peptide precursor *i*, write $p_{i}$ for the probability of detection. We modeled $p_{i}$ using logit regression splines of the form where the $x_{ik}$ are natural spline basis vectors computed by the ns() function in the splines package in R. The basis vectors were computed from the average observed log-intensities ${\bar{y}}_{\text{obs},i}$ for each precursor. The number of basis vectors is called the degrees of freedom (df) for the spline. Splines were fitted with df=1, 3, or 5. For df=3 and df=5 the spline knots were set according to the ns() function default, which separates the ordered ${\bar{y}}_{\text{obs},i}$ values into equal numbered groups. For df=1, the logit-linear model: was used as the regression spline. When fitting regression splines at the protein-level data, basis vectors were generated on the average observed log-intensity for each protein (or protein group).


$$\text{logit}{\;p}_{i}=\beta_{0}+\beta_{1}x_{i1}+\beta_{2}x_{i2}+\beta_{3}x_{i3}+\beta_{4}x_{i4}+\beta_{5}x_{i5},$$

$$\text{logit}{\;p}_{i}=\beta_{0}+\beta_{1}{\bar{y}}_{\text{obs},i},$$

### 2.3 Zero-truncated binomial distribution

The number of detected samples for each precursor was modeled by the zero-truncated binomial distribution, that is, *n* being the sample size and $d_{i}$ being the number of detected intensity values in precursor *i*. The probability of detecting a sample is precursor-specific with $p_{i}\in [0,1]$. The probability of detecting exactly *k* samples in precursor *i* is given by for k=1,2,…,n and $I_{p_{i}}(1,n)=1-{\left(1-p_{i}\right)}^{n}$. The spline regression parameters were estimated by maximum likelihood using the zero-truncated binomial distribution.


$$d_{i}∼\text{ZB}(n,p_{i}),$$

$$p(d_{i}=k)=\left(\begin{array}{c} n\\ k \end{array}\right)\frac{p_{i}^{k}{\left(1-p_{i}\right)}^{n-k}}{I_{p_{i}}(1,n)},$$

### 2.4 Capped logit-linear model

The capped logit-linear model adds an asymptote parameter to the logit-linear model. The model assumes that the probability of detection for precursor *i* is where ${\bar{y}}_{\text{obs},i}$ is the average observed log-intensity for that precursor. Here 0<α≤1 imposes a cap on the detection proportion for all precursors. Assuming that $\beta_{1}>0$, α is the asymptotic probability of detection for precursors with high observed intensities. The capped logit-linear model was estimated by maximizing the zero-truncated binomial likelihood with respect to the parameters $\beta_{0}$, $\beta_{1}$, and α. The same model was applied also to protein-level intensities, in which case ${\bar{y}}_{\text{obs},i}$ was the averaged observed log-intensity for protein *i*.


$$p_{i}=\frac{\alpha e^{\beta_{0}+\beta_{1}{\bar{y}}_{\text{obs},i}}}{1+e^{\beta_{0}+\beta_{1}{\bar{y}}_{\text{obs},i}}},$$

### 2.5 Simulation

Normally distributed log2-intensity values were generated for 10 000 proteins and 12 samples in two groups of 6. Protein-wise means varied from 5 to 12. The standard deviation between replicates was 0.3. Two-fold differential expression was generated for 1000 randomly selected proteins (with 500 in each direction). Missing values were generated according to a logit-linear DPC with parameters $\beta_{1}=0.8$ and $\beta_{0}=-6.0$. Missing values were imputed using methods implemented in the msImpute (Hediyeh-zadeh et al. 2020), MsCoreUtils (Lazar et al. 2016; Rainer et al. 2022), and Perseus (Tyanova et al. 2016) software packages. Differential expression analyses were run using limma (Ritchie et al. 2015) before and after missing value imputation with false discovery rate (FDR) <0.05 as the significance cutoff.

## 3 Results

### 3.1 Detection increases with intensity in proteomics data

We first demonstrate the relationship between missingness and observed intensity on the precursor level using a variety of previously published proteomics datasets. We focus on datasets consisting of two or more replicate samples (biological or technical replicates) for two or more experimental conditions. Each dataset therefore consists of a matrix of log-intensities $y_{ij}$ for peptide precursors i=1,…,m and samples j=1,…,n. If a particular $y_{ij}$ is non-missing, then we say that precursor *i* is *detected* in sample *j*. We will write $d_{ij}=1$ if $y_{ij}$ is detected and $d_{ij}=0$ if $y_{ij}$ is missing. We will further write $d_{i}=\sum_{j=1}^{n}d_{ij}$ for the total number of detected (non-missing) values for precursor *i*.

Our aim is to explore the probability of detection as a function of the intensity value $y_{ij}$, but we cannot do this directly because the intensities of non-detected precursors are unknown. Instead we take advantage of the fact that expression intensities typically vary by orders of magnitude across different peptide precursors but are relatively less variable across samples for the same precursor. For most datasets, it is reasonable to assume that the majority of precursors are not differentially expressed between conditions. Even for differentially expressed precursors, the expression fold-changes are typically less than one order of magnitude with only a minority of larger fold-changes. We therefore examine the proportion of missing values for each precursor as a function of the mean of the observed log-intensities for that precursor, ${\bar{y}}_{\text{obs},i}$, with the confidence that the observed log-intensities for each precursor are at least roughly representative of the likely magnitude of the unobserved intensities for the same precursor.

**Figure 1:**
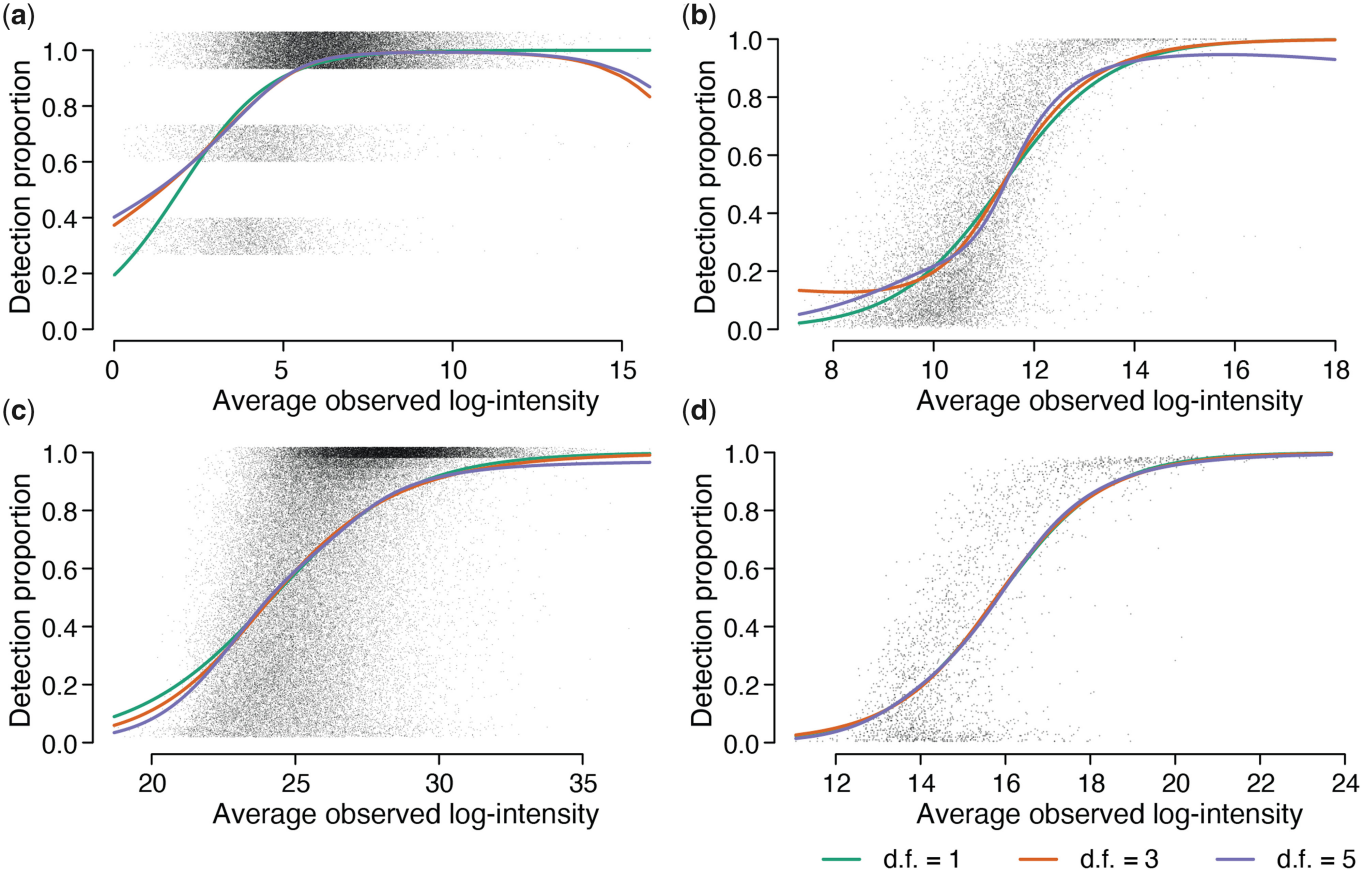
The proportion of detected values increases with the average intensity of each peptide precursor. Panels (a)–(d) show scatter plots for Datasets A–D. The *x*-axis shows average log2 observed intensity for each precursor. The *y*-axis shows the proportion of detected (non-missing) values for each precursor. The number of samples is (a) n=3, (b) n=231, (c) n=27, and (d) n=212. Jittering is added to the detection proportions in (a) and (c) to reduce over-plotting. Natural cubic splines were fitted to the logit proportions with 1, 3, or 5 degrees of freedom (df).

Figure 1 shows empirical logit spline curves fitted to the observed proportion of detected values for each peptide precursor for four public datasets. The proportion of detected samples increases as the average observed intensity increases on precursor-level data, regardless of the overall frequency of missing values in the dataset. The number of samples varies from n=3 to n=231 for the different datasets, but the increasing trend is discernible even for the smallest dataset. The same monotonic increasing relationship between average log-intensity and proportion detected was also observed when intensities were summarized at the protein level for the same datasets (Supplementary Fig. S1). Supplementary Figs S6 and S7 show the same increasing relationship for two more datasets. This strong relationship between intensity and detection proportion shows that the missing values do not occur at random but are probabilistically related to peptide expression.

### 3.2 The observed proportion of detected samples is zero-truncated

Precursors that are missing in all samples are omitted from Fig. 1 because there are no observed intensities from which to compute ${\bar{y}}_{\text{obs},i}$. In Fig. 1a, for example, the dataset contains n=3 samples and only precursors with 1, 2, or 3 observed intensities can be shown on the plot. The absence of precursors with zero detected values needs to be taken into account when estimating the detection proportion curves. We model the number of detected samples for each precursor by a zero-truncated binomial distribution, i.e. $d_{i}∼\text{ZB}(n,p_{i}),$ where $d_{i}$ is the number of detected samples for precursor *i*, *n* is the sample size, $p_{i}$ is the detection probability for that precursor and ZB denotes the zero-truncated binomial distribution. In Figure 1, we model $\text{logit}(p_{i})$ as a spline function of the average log-intensity $y_{\text{obs},i}$ and the parameters of the spline are estimated by maximum likelihood using the truncated binomial likelihood.

To demonstrate the effect of using the zero-truncated binomial distribution, we re-fitted the spline curves to Dataset A using both zero-truncated and standard binomial distributions. The detection probability is consistently overestimated at lower intensities if the ordinary binomial distribution is used (Supplementary Fig. S2). The overestimation remains regardless of the number of parameters used for the regression spline. The zero-truncation adjustment provides more accurate estimation of detection probabilities for lower intensity precursors especially when the sample size is limited.

### 3.3 The detection proportion is approximately logit-linear in log-intensity

The spline curves in Figure 1 were estimated with either 1, 3, or 5 degrees of freedom (df), with the df being equal to the number of regression coefficients estimated in the fit. For each dataset, the three fitted curves are not materially different, suggesting that the logit-linear curve, with 1 degree of freedom, is sufficient to summarize the intensity-dependent trend. To explore this more rigorously, we computed the percentage of deviance explained by each regression spline coefficient. We defined the total deviance to be twice the log-likelihood difference between most complex spline regression model with 5 df and the null model with only an intercept term. The deviance explained by each spline degree of freedom can then be evaluated by increases in the log-likelihood as parameters are added to the spline curve. We found that almost all of the log-likelihood difference was explained by the linear coefficient (Table 1). The logit-linear coefficient explains over 96% of the total deviance for all datasets. The four non-linear parameters together explain <4% of the deviance. At the protein-level, the logit-linear trend explains over 97% of the deviance (Supplementary Table S1). Similar results were observed for datasets E and F (Supplementary Figs S6c and S7c). These results suggest that the probability of detection for each precursor (or protein) is approximately linear in log-intensity on the logit scale. We will therefore assume that the detection probability can be represented as a logit-linear function of log-intensity for the remainder of this article.

**Table 1.** Percentage of total deviance explained by the logit-linear and non-linear regression splines in Fig. 1.^a^

	Deviance explained (%)			
Source	A	B	C	D
Linear	97.242	96.40	99.438	99.896
Non-linear 2–3	2.732	2.32	0.328	0.029
Non-linear 4–5	0.026	1.28	0.234	0.075

a The rows of the table show the percentage of the total deviance explained by the logit-linear curve and the additional deviance explained by logit splines with 3 or 5 df. The second row gives the additional deviance explained by df =3 over df =1 and the third row the additional deviance explained by df =5 over df =3. The logit-linear curve explains >96% of the deviance for all datasets.

### 3.4 Missingness is negligible for high intensity precursors

The detection proportion curves shown in Figure 1 approach 100% for high observed intensities. If some of the missing values occur for reasons that are unrelated to the intensity level, then a proportion of missing values should persist even for very high intensity precursors. To explore whether this is true, we extended the logit-linear model to allow the curve to asymptote at a value <100%. The DPCs were found to asymptote at values very close to 1 for all datasets (Figure S3). The residual proportion of missing values for large intensities is always <3% and usually <1%. This shows that missing value mechanisms that are unrelated to intensity must be limited to a very small proportion of precursors. The same phenomenon is observed for intensities summarized at the protein-level (Supplementary Fig. S4) and for Supplementary Datasets E and F (Supplementary Figs S6 and S7).

### 3.5 A formal model for the detection probabilities

So far we have examined empirically the relationship between missingness and intensity, leveraging the observed data. We now propose a formal model for the detection probabilities that allows us to estimate the probability that an observation is detected (non-missing) given its own underlying intensity, even when that underlying intensity is not observed.

As before, let $y_{ij}$ be a log-intensity value and let $d_{ij}$ indicate detection or not for that value. If $d_{ij}=0$, then $y_{ij}$ represents the intensity that would have been returned if the missingness mechanism had been absent or had not operated. Motivated by the results of the previous section, we assume a logit-linear relationship between the detection probability and the underlying intensity, where $p(d_{ij}=1|y_{ij})$ is the probability of detection conditional on $y_{ij}$. We expect $\beta_{1}>0$ as higher intensity values are more likely to be detected. The detection probabilities are precursor- and sample-specific, but the coefficients $\beta_{0}$ and $\beta_{1}$ are assumed to be global across all precursors and samples.


(1) $$\text{logit}\;p(d_{ij}=1|y_{ij})=\beta_{0}+\beta_{1}y_{ij},$$

### 3.6 Inferring the missing value distribution

For simplicity of notation, we will drop the subscripts *ij* for the next three sections, although our discussion is still for a specific precursor and sample. Write $f_{\text{obs}}(y)=f(y|d=1)$ for the observed data distribution, i.e. the probability distribution for *y* conditional on *y* being observed. Similarly write $f_{\text{mis}}(y)=f(y|d=0)$ for the missing data distribution, i.e. the probability distribution for *y* conditional on *y* being unobserved.

We can estimate $f_{\text{obs}}(y)$ from the observed data, so the key question is what can be said about the $f_{\text{mis}}(y)$? It turns out that the detection probability formula (1) allows us to relate the missing value distribution to the observed value distribution through a mathematical process called *exponential tilting* (Kim and Yu 2011). Application of Bayes theorem gives


$$\frac{f_{\text{mis}}(y)}{f_{\text{obs}}(y)}=\frac{p(d=0|y)}{p(d=1|y)}\frac{p(d=1)}{p(d=0)}.$$

Substituting in the detection probabilities allows us to conclude that


(2) $$f_{\text{mis}}(y)=\frac{p(d=1)}{p(d=0)}\text{exp}(-\beta_{0}-\beta_{1}y)f_{\text{obs}}(y).$$

Applying the definition of moment generating functions further shows that where $M_{\text{obs}}$ is the moment generating function of the observed distribution. The distribution of the unobserved values is an exponentially tilted transform of the observed distribution.


(3) $$f_{\text{mis}}(y)=\frac{e^{-\beta_{1}y}}{M_{\text{obs}}(-\beta_{1})}f_{\text{obs}}(y),$$

It is natural to assume that $f_{\text{obs}}(y)$ is normal, i.e. the observed intensities follow a normal distribution with mean $\mu_{\text{obs}}$ and variance $\sigma_{\text{obs}}^{2}$. It follows from exponential tilting (3) that the unobserved intensities must also follow a normal distribution with the same variance but a decreased mean. Specifically, y|d=0 follows a normal distribution with variance $\sigma_{\text{mis}}^{2}=\sigma_{\text{obs}}^{2}$ and mean


$$\mu_{\text{mis}}=\mu_{\text{obs}}-\beta_{1}\sigma_{\text{obs}}^{2}.$$

This reveals that the separation between the observed and missing data distributions depends on the slope $\beta_{1}$ of the logit-linear DPC (1). The quantity $\beta_{1}\sigma_{\text{obs}}$ is scale-invariant and represents the number of standard deviations by which the missing values are biased relative to the observed values. A complete mathematical proof of this result is given in Supplementary Methods Section S1.4.

### 3.7 Correcting the DPC for bias

For the purpose of estimating $\beta_{0}$ and $\beta_{1}$, we need the marginal detection probability not conditional on a possibly unobserved intensity value. By substituting the expression (3) for $f_{\text{mis}}(y)$ into equation (2), the marginal log-odds of detection can be written as or equivalently


$$\text{logit}\;p(d=1)=\beta_{0}+\beta_{1}(\mu_{\text{mis}}+\mu_{\text{obs}})/2.$$

(4) $$\text{logit}\;p(d=1)=\beta_{0}+\beta_{1}\mu_{\text{obs}}-\frac{1}{2}\beta_{1}^{2}\sigma_{\text{obs}}^{2},$$

This formula defines the marginal DPC entirely in terms of the observed probability distribution. The probability of detection depends only on the global coefficients $\beta_{0}$ and $\beta_{1}$ and on precursor-wise quantities $\mu_{\text{obs}}$ and $\sigma_{\text{obs}}^{2}$ that can be estimated from the observed intensities.

To estimate $\beta_{0}$ and $\beta_{1}$, we take advantage of the fact that, for a well-designed experiment, most precursors will not be differentially expressed between experimental conditions. It is therefore reasonable to assume that $\mu_{\text{obs}}$, $\sigma_{\text{obs}}^{2}$, and p(d=1) are precursor-specific but not sample-specific. We can therefore substitute in the observed precursor means and variances for $\mu_{\text{obs}}$ and $\sigma_{\text{obs}}^{2}$ and then estimate $\beta_{0}$ and $\beta_{1}$ from the precursor-level detection proportions.

**Figure 2:**
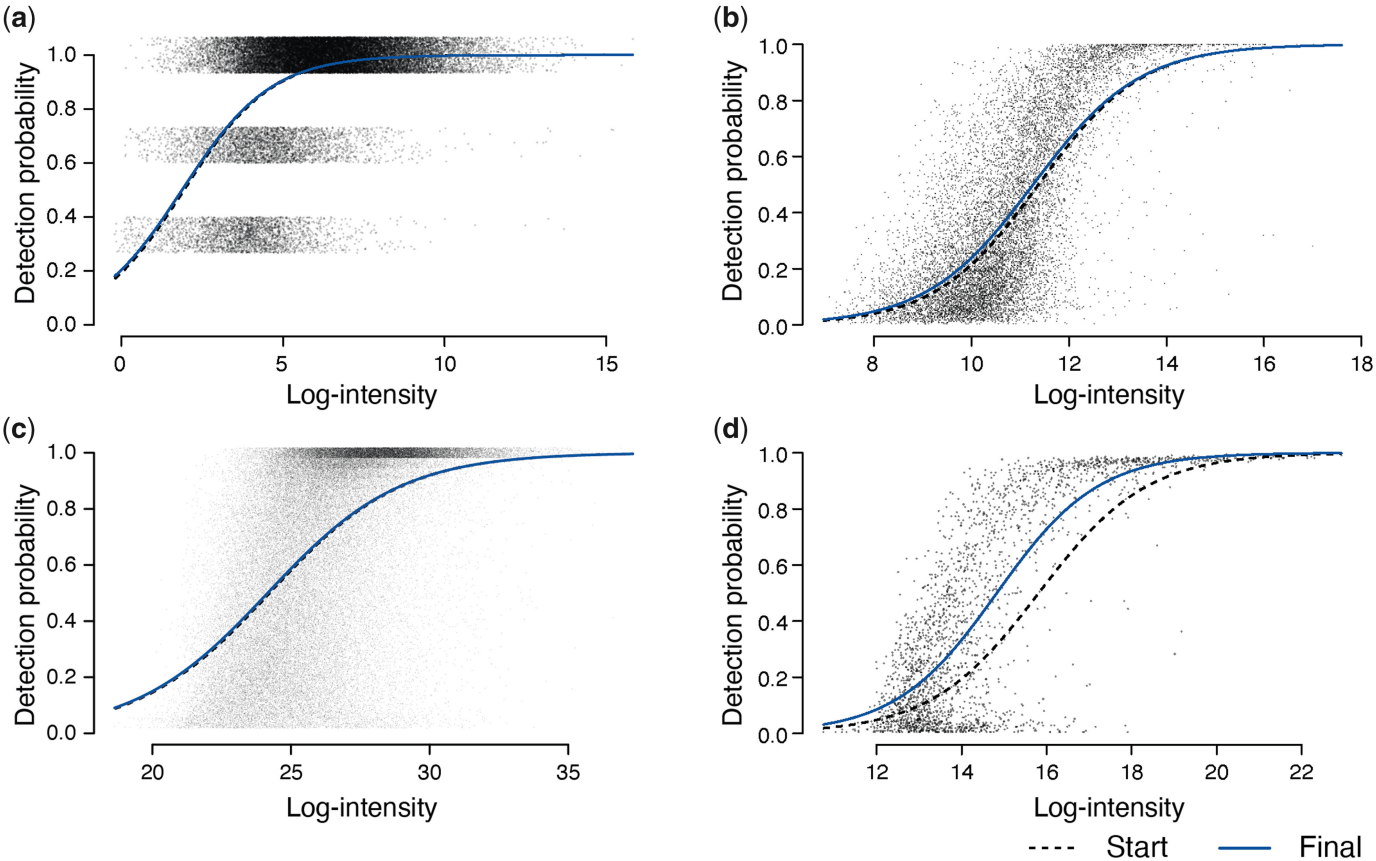
DPCs. Panels (a)–(d) show estimated DPCs for Datasets A–D at the precursor level. In each dataset, the starting curve is obtained by fitting a logistic linear curve for detection proportions to average observed intensities whereas the final curve relates detection probabilities to underlying log-intensities. Jittering is added to vertical axes in (a) and (c) to reduce over-plotting in precursors. The *x*-axis here is $(\mu_{\text{mis}}+\mu_{\text{obs}})/2$, which is the precursor mean log-intensity with observed and missing intensities equally weighted. The estimated curve parameters are given in Table 2.

Figure 2 shows the DPCs estimated for Datasets A–D. For each precursor, $\mu_{\text{obs}}$ is estimated by the average observed log-intensity and the variance parameter $\sigma_{\text{obs}}^{2}$ is estimated as the empirical Bayes moderated sample variance. Logistic regression coefficients $\beta_{0}$ and $\beta_{1}$ are estimated by maximum likelihood estimation assuming the zero-truncated binomial distribution for counts of observed samples in each precursor. The *x*-axis is $(\mu_{\text{mis}}+\mu_{\text{obs}})/2$, which represents the precursor mean log-intensity with observed and missing intensities equally weighted. The estimated $\beta_{0}$ and $\beta_{1}$ parameter values are shown in Table 2. The DPC generally reaches each detection probability at a slightly lower log-intensity once underlying intensities are taken into account because the observed intensities are biased slightly toward higher values for each peptide precursor.

**Table 2.** Parameter estimates for DPCs fitted on Datasets A–D that are visualized in Fig. 2.^a^

	DPC		
Dataset	Fitted on	$\beta_{0}$	$\beta_{1}$
A	Observed	−1.4264	0.7363
	Underlying	−1.3612	0.7258
B	Observed	−10.7271	0.9430
	Underlying	−10.3113	0.9154
C	Observed	−10.1931	0.4212
	Underlying	−10.0860	0.4176
D	Observed	−12.4087	0.7847
	Underlying	−12.4435	0.8393

a The lower (Underlying) values are the final estimates that related probability of detection to underlying log-intensity. The upper (Observed) values are the more naive estimates that are obtained by fitting a logit-linear trend to the observed log-intensities for each precursor.

### 3.8 Quantifying the information content of missing value occurrences

Since the missing value process is intensity-dependent, it follows that the frequency of missing values for a peptide or protein contains information about the expression level of that feature. Similarly, differences in the frequency of missing values between experimental conditions provides some evidence in support of differential expression. The DPC probability model developed in the previous section allows this information to be quantified. Consider a set of *n* replicate samples and write p=p(d=1) for the detection probability defined by Equation (4) for a particular peptide or protein. On average, we expect *np* observed intensities and n(1−p) missing values. The mean intensity $\mu_{\text{obs}}$ can be estimated from the arithmetic average of the observed intensities. Alternatively, $\mu_{\text{obs}}$ could also be estimated from the proportion $\hat{p}$ of observed values by $\left(\text{logit}(\hat{p})-\beta_{0}+\beta_{1}^{2}\sigma_{\text{obs}}^{2}/2\right)/\beta_{1}$. If *np* values are observed, then the Fisher information for $\mu_{\text{obs}}$ from the observed intensities is $I_{1}=np/\sigma_{\text{obs}}^{2}$. In contrast, the Fisher information for $\mu_{\text{obs}}$ from the missing value frequency is $I_{2}=n\beta_{1}^{2}p(1-p)$. This formula follows from the fact that *p* is logit-linear in $\mu_{\text{obs}}$ with coefficient $\beta_{1}$. The ratio of the Fisher informations from the two sources is $I_{2}/I_{1}=\beta_{1}^{2}(1-p)\sigma_{\text{obs}}^{2}$. Considering that $\beta_{1}^{2}(1-p)$ is almost always <1 and that $\sigma_{\text{obs}}^{2}$ is typically estimated to be around 0.1, it follows that the information that can be inferred from the missing value occurrences is many times smaller than the information arising from the observed intensities themselves.

### 3.9 Application to differential expression

The DPC can be used to directly model the likelihood of missing values without the need for imputation. We simulated log2-intensities for 10 000 proteins in two experimental groups with n=6 samples in each group. Two-fold expression changes were simulated for 1000 randomly chosen proteins while other proteins were not differentially expressed. Missing values were introduced according to a logit-linear DPC with parameters similar to those estimated from read data (Supplementary Fig. S8). The estimated DPC agreed almost exactly with the true curve (${\hat{\beta }}_{0}=-6.02$, ${\hat{\beta }}_{1}=0.801$).

**Figure 3:**
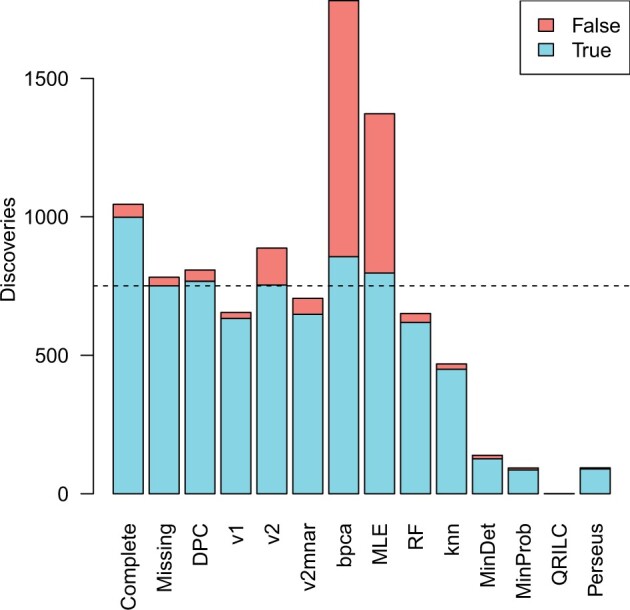
Differential expression performance. The average number of true positives and false discoveries achieved by various missing value methods when assessing differential expression between two groups. limma was used for all analyses except “DPC.” Results are averaged over 10 simulated datasets. “Complete” gives results from full data without missing values. “Missing” gives results with missing values left in the data, and the number of true positives found by this method is marked by a horizontal dotted line. “DPC” gives results from likelihood ratio tests using the DPC. “v1,” “v2,” and “v2mnar” are imputation methods implemented in the msImpute package. “Perseus” is the imputation strategy used by Perseus software. The remaining are imputation methods implemented by the MSnbase and MsCoreUtils packages.

A limma differential expression analysis on the complete data (without missing values) achieved a very high true positive rate (TPR) of 99.85% while controlling the FDR below 5% (Figure 3, Supplementary Table S4). After introducing missing values, the TPR of the limma analysis dropped to 75%, reflecting the loss of information when observations are removed.

Likelihood ratio tests for differential expression using the DPC achieved a modest increase in the TPR to 76.72% while keeping the FDR at 5%. In this approach, the likelihood of a missing value was $\text{log}\;p(d_{ij}=0;\mu_{ij},\sigma^{2})$ where $p(d=0;\mu ,\sigma^{2})=\int p(y;\mu ,\sigma^{2})p(d=0|y,\beta_{0},\beta_{1})dy$ was evaluated by Gaussian quadrature (Golub and Welsch 1969) implemented in the gauss.quad function of the statmod software package (https://cran.r-project.org/package=statmod).

In contrast, imputation methods performed poorly. The imputation methods bpca, MLE and v2 returned high numbers of significant results but at the cost of unacceptably high FDRs. All other imputation methods returned fewer true positives than the simple limma analysis with missing values.

## 4 Discussion and conclusion

It has been observed previously that lower intensity peptides tend to yield more missing values. Here we demonstrate that this is a universal phenomenon for label-free shotgun proteomics. The trend is seen for both protein and precursor-level data and for datasets with high or low overall rates of missingness. We show that the DPC can be accurately modeled as a logit-linear function of the underlying log-intensity. We also show that the proportion of missing values becomes negligible for peptide precursors or proteins with sufficiently high intensities.

There has been much discussion in the literature about the relevance of Donald Rubin’s missing at random (MAR) and missing not at random (MNAR) classification for mass spectrometry data (Karpievitch et al. 2012; Webb-Robertson et al. 2015; Lazar et al. 2016; Wang et al. 2020; Gardner and Freitas 2021; Liu and Dongre 2021; Dekermanjian et al. 2022; Shen et al. 2022). Our work shows that missing intensities are MNAR but that the dependence of missing value frequency on intensity is gradual.

The most common MNAR model is to assume that missing values are left-censored (Karpievitch et al. 2009, 2012; Tekwe et al. 2012; Wieczorek et al. 2017). Our work unifies the MAR and left-censoring models into one. Our logit-linear DPC is equivalent to MAR if the slope $\beta_{1}$ is close to zero (making the DPC a flat line) and is equivalent to left-censoring if $\beta_{1}$ is very large (making the DPC a step function). Our work shows that neither of these extremes are compatible with real data and a gradual DPC with $\beta_{1}\approx 1$ is a better representation. More generally, the slope $\beta_{1}$ of the DPC quantifies the amount of information that can be theoretically extracted from the missing value frequencies for estimating peptide intensities or assessing differential expression. Some authors have proposed classifying individual missing values as MAR or left-censored (Lazar et al. 2016; Wei et al. 2018; Liu and Dongre 2021; Dekermanjian et al. 2022) but our work shows that the same DPC can be applied to all values.

We show that the mathematical concept of exponential tilting applies to mass spectrometry data, allowing the distribution of the missing (unobserved) intensities to be inferred from the observed values. When estimating the true DPC, we use the zero-truncated binomial distribution to avoid observational bias on the y-axis and exponential tilting to avoid observational bias on the *x*-axis. We provide an R software package that implements our estimation procedures. Our DPC method can be applied to new datasets to quantify the statistical properties of the missing value process.

The estimated DPC provides an accurate probability model for the missing value occurrences that can potentially be used in a number of ways at either the peptide or protein level. One direct approach is to model the likelihood of missing values as a function of unknown linear model parameters as part of a likelihood-based analysis. Such an approach has the advantage of fully leveraging all the statistical information that is contained in the observed intensities and the missing value frequencies together, while also avoiding the need for imputation. We showed that likelihood ratio tests using the DPC were able to recover more information in a differential expression analysis compared to a standard limma linear modeling approach in which missing values are dropped for each feature. The gain was modest but worthwhile, while imputation methods not using the DPC all performed poorly. The DPC was the only approach to recover more true discoveries than a limma analysis with NAs while controlling the FDR at an acceptable level.

## Supplementary Material

btad200_Supplementary_Data

## Data Availability

All datasets analyzed in this article are publicly available as described in Section 2.
