# Supplementary material for: Neither random nor censored: estimating intensity-dependent probabilities for missing values in label-free proteomics
Source: Bioinformatics. 2023 Apr 17;39(5):btad200. doi: 10.1093/bioinformatics/btad200 (PMC10174703; doi:10.1093/bioinformatics/btad200)
Supplement: btad200_Supplementary_Data [file btad200_supplementary_data.pdf]

Supplementary materials for

Missing values are informative in label-free shotgun proteomics data: estimating the detection probability curve

Mengbo Li [1,2], **Gordon K. Smyth** [1,3*]

[1]Bioinformatics Division, The Walter and Eliza Hall Institute of Medical Research, 1G Royal Parade, Parkville, Victoria 3052, Australia
[2]Department of Medical Biology, The University of Melbourne, Parkville, Victoria 3010, Australia
[3]School of Mathematics and Statistics, The University of Melbourne, Parkville, Victoria 3010, Australia

[*]To whom correspondence should be addressed. Tel: +61 3 9345 2326; Fax: +61 3 9347 0852; Email: smyth@wehi.edu.au

1 Supplementary Methods

1.1 Protein-level intensities for Datasets A-D

The missingness-intensity relationship was also examined for Datasets A-D on the protein group level. For the two DIA-NN [1] processed datasets, that is, Datasets A and B, MaxLFQ [2] intensities were obtained from their respective DIA-NN reports. For the two MaxQuant processed datasets [3], i.e., Datasets C and D, the `proteinGroups.txt` tables output by the MaxQuant software were used as the protein group level summary. The overall proportions of missing data are respectively 5.0%, 47.4%, 5.5% and 41.2% in Datasets A, B, C and D. The numbers of protein groups detected in at least one sample from the corresponding dataset are respectively $5,974$; $2,661$; $6,282$ and 332 for Datasets A-D.

1.2 Dataset E: Sydney Heart Bank

Cryopreserved left ventricular myocardium samples were analysed from donor hearts procured but not used for heart transplantation [4]. Mass spectrometry (MS) data were acquired in data-independent acquisition (DIA) mode and analysed by Spectronaunt v12, using a spectral library generated from fractions of the pooled mixture of all samples and analysed by LC-MS/MS in data-dependent acquisition (DDA) mode. Details on sample preparation, LC-MS/MS experiment workflow and data processing are available in [4]. Here we consider the healthy donor heart samples. Both precursor- and protein group-level data are log2-transformed before analysis. For the precursor-level analysis, there are $42,742$ precursors detected in at least one of the 24 samples with an overall missingness proportion of approximately 33.4%. To obtain protein-level intensities, MaxLFQ [2] was applied. In protein-level data, the proportion of missing data is about 12.0% in $3,208$ proteins. The dataset is publicly available from the ProteomeXchange Consortium via the PRIDE [5] partner repository with the dataset identifier PXD018678.

1.3 Dataset F: UPS1 spiked-in yeast extract

Three concentrations of UPS1 (25 fmol, 10 fmol and 5 fmol) were spiked in yeast extract in triplicates [6]. MS data were obtained in DDA mode and processed by MaxQuant [3]. Here we look at the dataset that compares 25 fmol to 10 fmol spiked-ins. Processed data were downloaded from ProteomeXchange Consortium via the PRIDE [5] partner repository with the dataset identifier PXD002370. For peptide-level data, we used the `peptides.txt` file and for the protein-level analysis, we used the `proteinGroups.txt` file from the MaxQuant output. Log2-transformation was applied to precursor- and protein group-level intensities. In the peptide-level data, we observe $13,186$ peptide species detected in at least one of the 6 samples. The missingness proportion is 14.7%. In the protein group-level data, we observe an overall proportion of 6.8% missing values and $2,342$ protein groups.

1.4 Mathematical derivation of detection probability curve

1.4.1 Observed and missing distributions

Let y be a log-intensity value and d be the indicator of detection with $d = 1$ if y is observed and $d = 0$ if y is missing. Write $f_{\text{obs}}(y) = f(y|d = 1)$ for the observed data distribution, i.e., the probability distribution for y conditional on y being observed. Similarly write $f_{\text{mis}}(y) = f(y|d = 0)$ for the missing data distribution, i.e., the probability distribution for y conditional on y being unobserved.

It follows from Bayes theorem that

$$f_{\text{obs}}(y) = f(y|d=1) = \frac{p(d=1|y)f(y)}{p(d=1)}$$

and

$$f_{\text{mis}}(y) = f(y|d=0) = \frac{p(d=0|y)f(y)}{p(d=0)}.$$

where $f(y)$ is the marginal density distribution of y, $p(d=1|y)$ is conditional detection probability, $p(d=1)$ is the marginal detection probability and $p(d=0) = 1 - p(d=1)$. The ratio of the missing to observed density functions is therefore related to the detection probabilities by

$$\frac{f_{\text{mis}}(y)}{f_{\text{obs}}(y)} = \frac{p(d=0|y)}{p(d=1|y)}\frac{p(d=1)}{p(d=0)}.$$

1.4.2 Assume logit-linear detection probabilities

We assume that the detection probability is a logit-linear function of y,

$$\operatorname{logit} p(d=1|y) = \beta_0 + \beta_1 y.$$

It follows that

$$\frac{f_{\text{mis}}(y)}{f_{\text{obs}}(y)} = \exp(-\beta_0 - \beta_1 y)\frac{p(d=1)}{p(d=0)}$$

which implies the missing value density is related to the observed value density by

$$f_{\text{mis}}(y) = \frac{p(d=1)}{p(d=0)}\exp(-\beta_0 - \beta_1 y) f_{\text{obs}}(y)$$

We know that the right-hand-side must integrate to 1. Also by definition

$$\int \exp(-\beta_1 y) f_{\text{obs}}(y) dy = M_{\text{obs}}(-\beta_1)$$

where $M_{\text{obs}}()$ is the moment-generating function of the observed distribution. Hence we can conclude that

$$M_{\text{obs}}(-\beta_1) = \exp(\beta_0)\frac{p(d=0)}{p(d=1)}$$

and therefore

$$f_{\text{mis}}(y) = \frac{e^{-\beta_1 y}}{M_{\text{obs}}(-\beta_1)} f_{\text{obs}}(y).$$

1.4.3 Assume observed values are normal

Let us assume now that the observed values follow a normal distribution, i.e., $f_{\text{obs}}(y)$ is a normal density with mean μ_{obs} and variance σ^2_{obs}. The normal moment generating function is

$$M_{\text{obs}}(-\beta_1) = \exp(-\beta_1 \mu_{\text{obs}} + \frac{1}{2}\beta_1^2 \sigma^2_{\text{obs}})$$

so

$$f_{\text{mis}}(y) = \exp\left(\beta_1 \mu_{\text{obs}} - \frac{1}{2}\beta_1^2 \sigma^2_{\text{obs}} - \beta_1 y\right)\left(2\pi\sigma^2_{\text{obs}}\right)^{-1/2} \exp\left\{-\frac{(y-\mu_{\text{obs}})^2}{2\sigma^2_{\text{obs}}}\right\}$$

which is the density of a normal distribution with mean

$$\mu_{\text{mis}} = \mu_{\text{obs}} - \beta_1 \sigma^2_{\text{obs}}$$

and variance

$$\sigma^2_{\text{mis}} = \sigma^2_{\text{obs}}.$$

1.4.4 Marginal log-odds of detection

The marginal log-odds of detection can be written as

$$\begin{aligned}
\log \frac{p(d=1)}{p(d=0)} &= \log \frac{\exp(\beta_0)}{M_{\text{obs}}(-\beta_1)} \\
&= \beta_0 + \beta_1 \mu_{\text{obs}} - \frac{1}{2}\beta_1^2 \sigma_{\text{obs}}^2 \\
&= \beta_0 + \beta_1 \frac{\mu_{\text{obs}} + \mu_{\text{mis}}}{2}.
\end{aligned}$$

2 Supplementary Figures

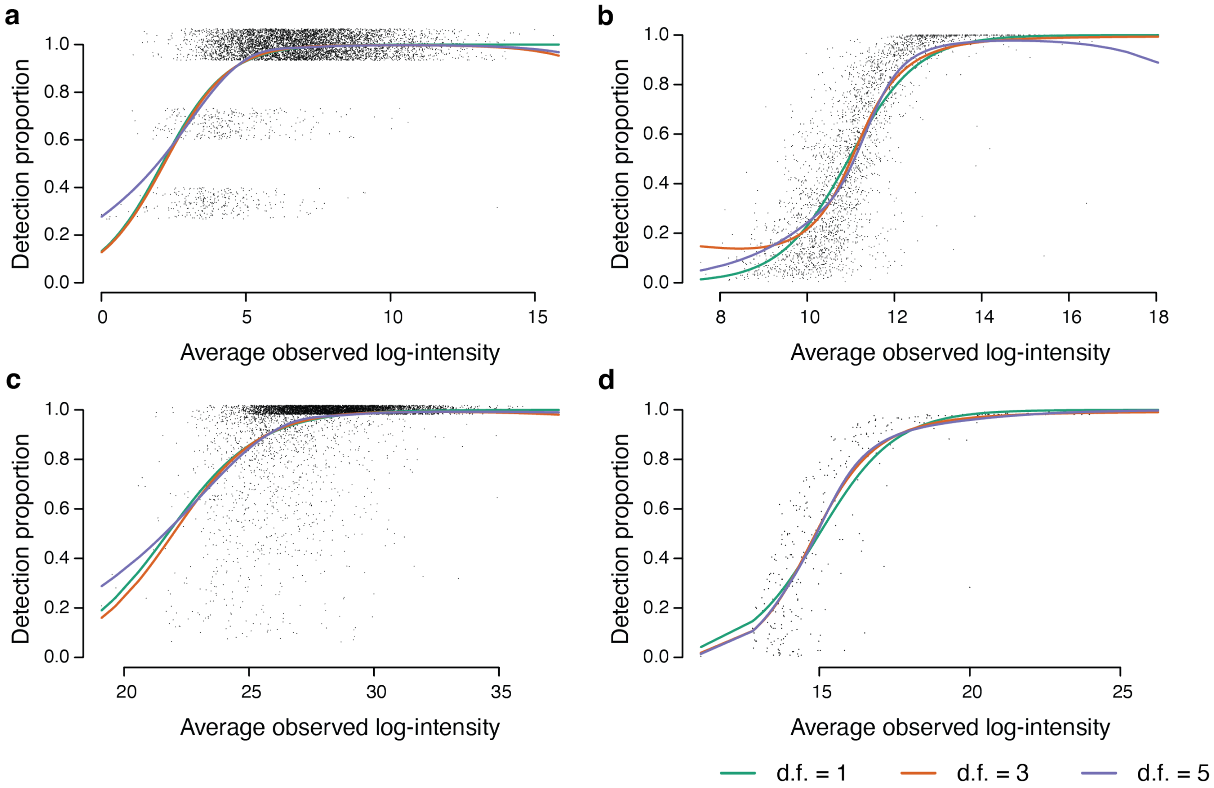

Figure S1: The proportion of detected samples increases in average intensity in each protein group. The x-axis shows the average observed intensity and the y-axis shows the proportion of detected samples in each protein group. Regression splines are fitted on the protein group-level for Datasets A–D in panels (a)–(d). Jittering is added to detection proportions in (a) and (c) to reduce over-plotting.

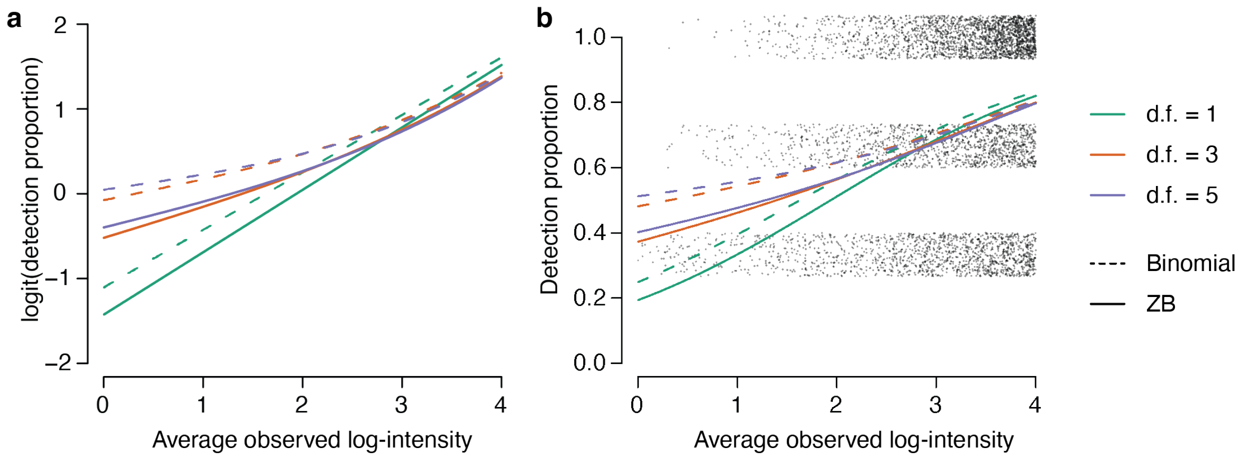

Figure S2: Detection probabilities are biased if zero-truncation is ignored. Nature regression splines are fitted to the Dataset A logit detection proportions by maximizing either the ordinary binomial likelihood (dashes lines) or the zero-truncated binomial likelihood (solid lines) on the precursor level. The splines use either 1, 3 or 5 df (green, red and black respectively). Panel (a) plots detection proportions on the logit scale and panel (b) shows untransformed proportions. Jittering is added to the detection proportions in (b) to reduce over-plotting. The detection probabilities are consistently over-estimated by the ordinary binomial likelihood.

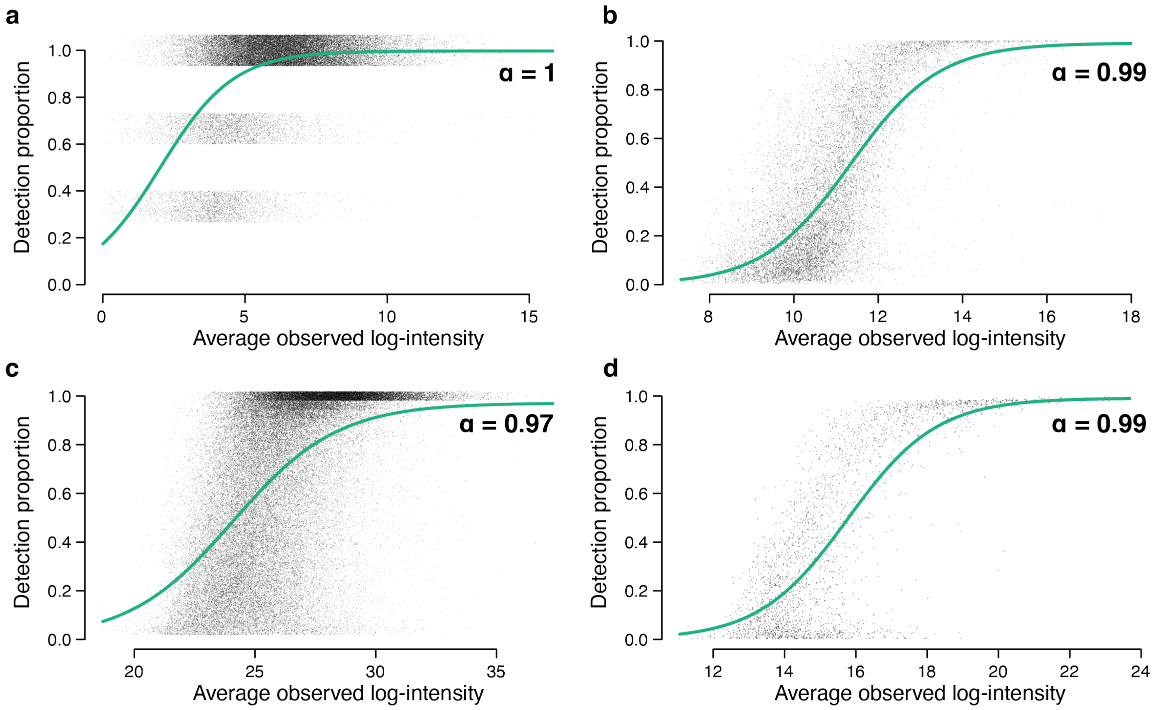

Figure S3: Capped logistic-linear curves for detection proportion on the precursor level. A logistic linear model is fitted to detection proportion on average observed intensity for Datasets A-D in (a)-(d) on the precursor level. Detection proportions for all observations are capped by α, where $\alpha \in (0, 1]$. The maximum likelihood estimates for the asymptotic probability (α) are (a) 0.9974, (b) 0.9915, (c) 0.9713 and (d) 0.9912. Jittering is added to vertical axes of (a) and (c) to reduce over-plotting in precursors.

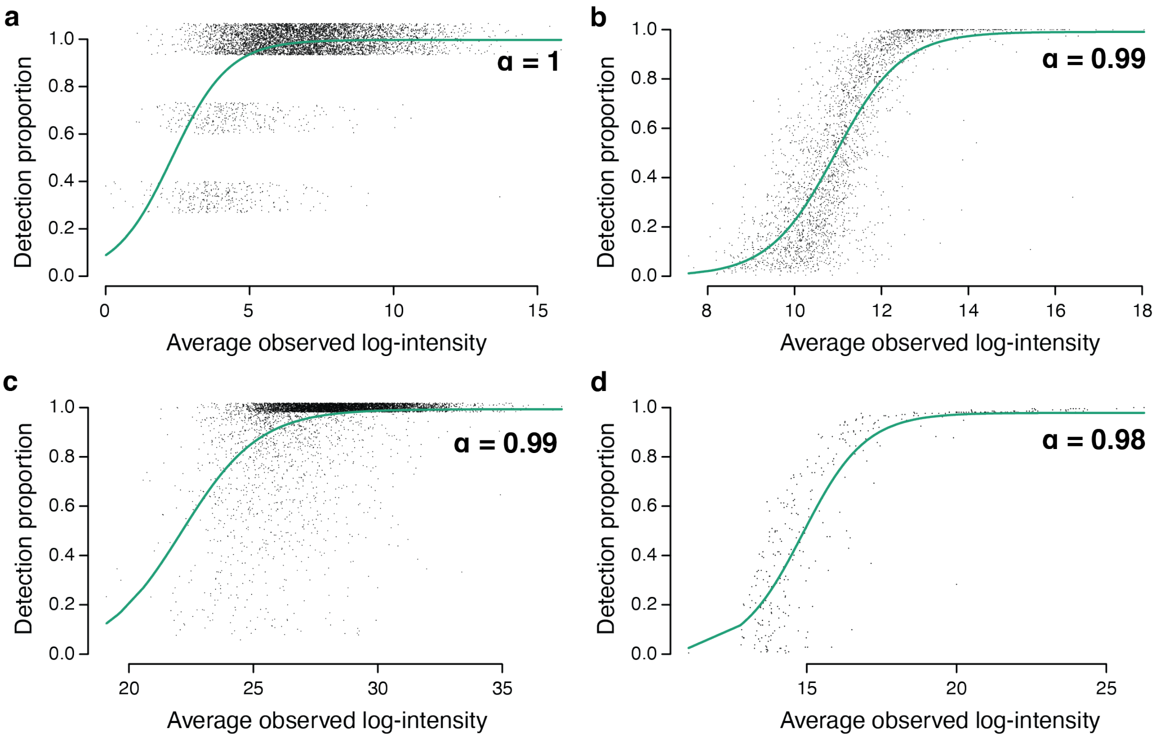

Figure S4: Capped logistic-linear curves for detection proportion on the protein group level. A logistic linear model is fitted to detection proportion on average observed intensity in each protein group for the Datasets A–D in (a)–(d). Detection proportions for all observations in the dataset are capped at $\alpha \in (0, 1]$. The maximum likelihood estimate of α for each dataset is indicated in (a)–(d). Jittering is added to vertical axes of (a) and (c) to reduce over-plotting in protein groups.

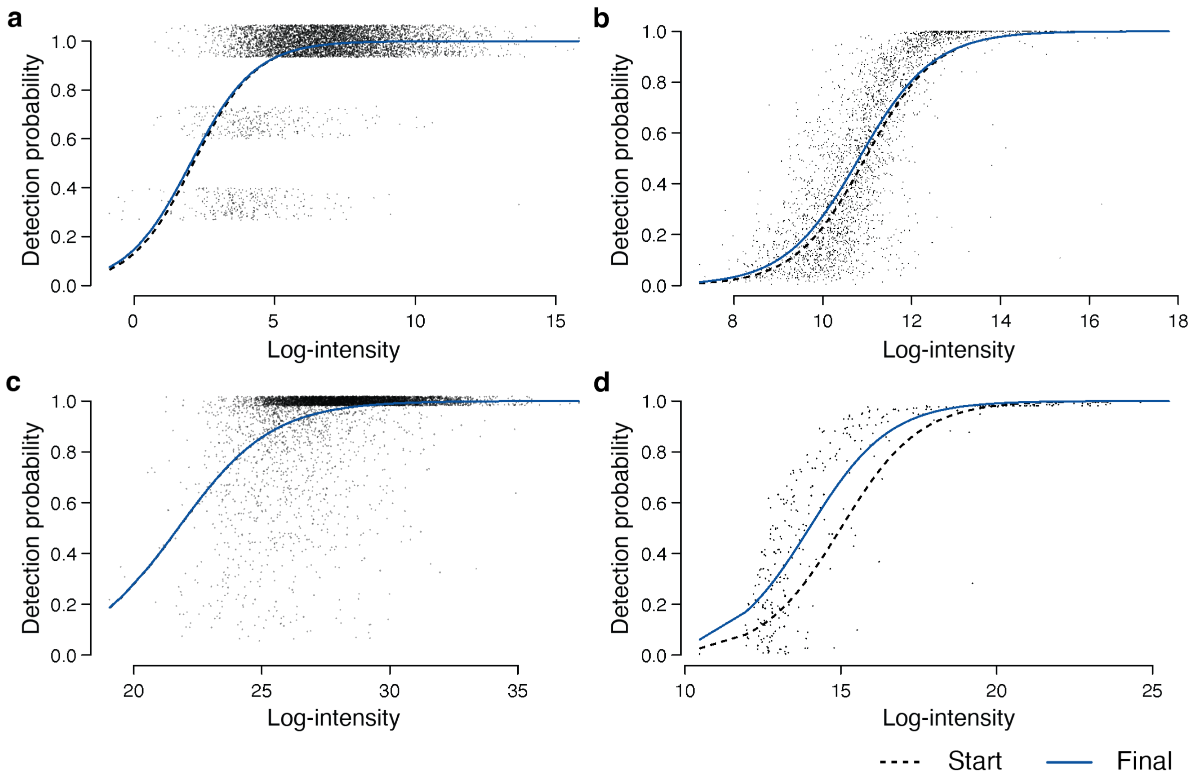

Figure S5: Detection probability curves fitted on the protein group level for Datasets A–D in (a)–(d). The starting curve is obtained by fitting a logistic linear curve for detection proportions to the average observed intensities on the protein group level. Jittering is added to vertical axes in (a) and (c) to reduce over-plotting. The estimated parameters for each curve are displayed in Table S2.

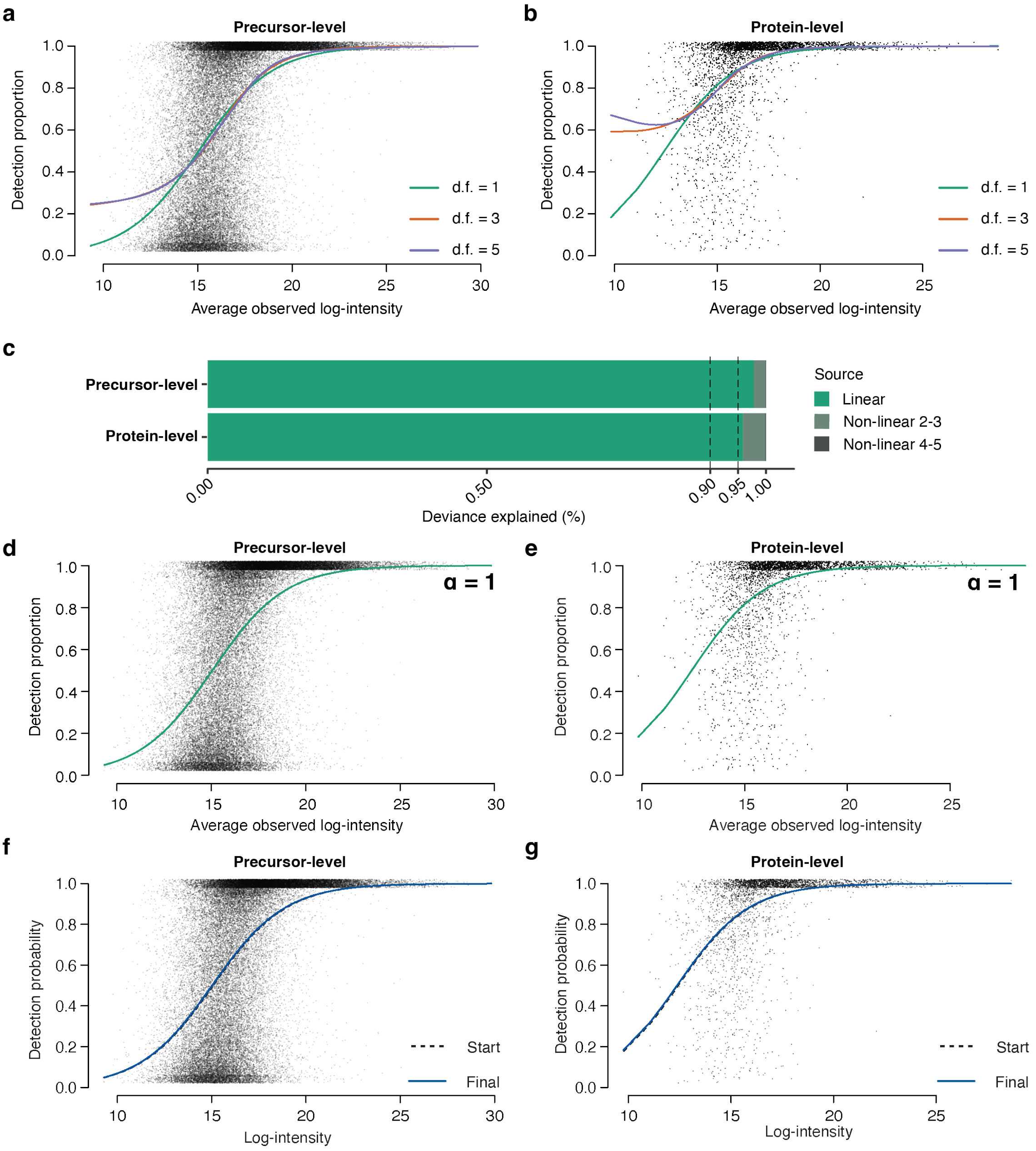

Figure S6: Relationship between missingness and intensity in Sydney Heart Bank data (Dataset E) on both precursor and protein group levels. (a) Regression splines fitted for detection proportions to average observed intensities in precursors. (b) Regression splines fitted for detection proportions to average observed intensities in protein groups. (c) Percentage of total reduced deviance explained by the logit-linear and non-linear regression splines from panels a and b. (d) Capped logistic-linear curve fitted for detection proportions to average observed intensities in precursors. (e) Capped logistic-linear curve fitted for detection proportions to average observed intensities in protein groups. (f) Detection probability curve fitted on the precursor level. (g) Detection probability curve fitted on the protein group level.

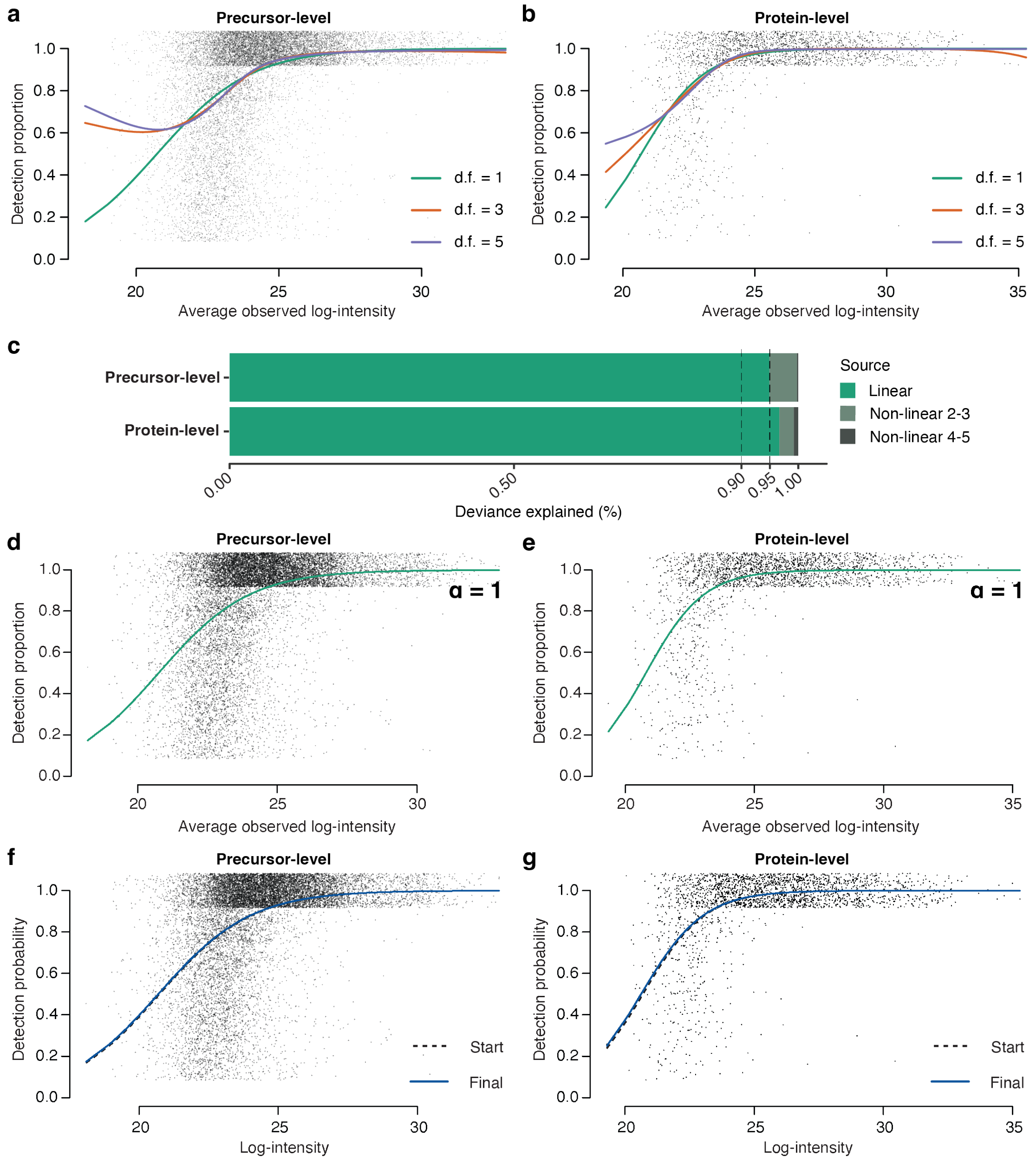

Figure S7: Relationship between missingness and intensity in UPS1 spiked-in yeast extract data (Dataset F) on both precursor and protein group levels. (a) Regression splines fitted for detection proportions to average observed intensities in precursors. (b) Regression splines fitted for detection proportions to average observed intensities in protein groups. (c) Percentage of total reduced deviance explained by the logit-linear and non-linear regression splines from panels a and b. (d) Capped logistic-linear curve fitted for detection proportions to average observed intensities in precursors. (e) Capped logistic-linear curve fitted for detection proportions to average observed intensities in protein groups. (f) Detection probability curve fitted on the precursor level. (g) Detection probability curve fitted on the protein group level.

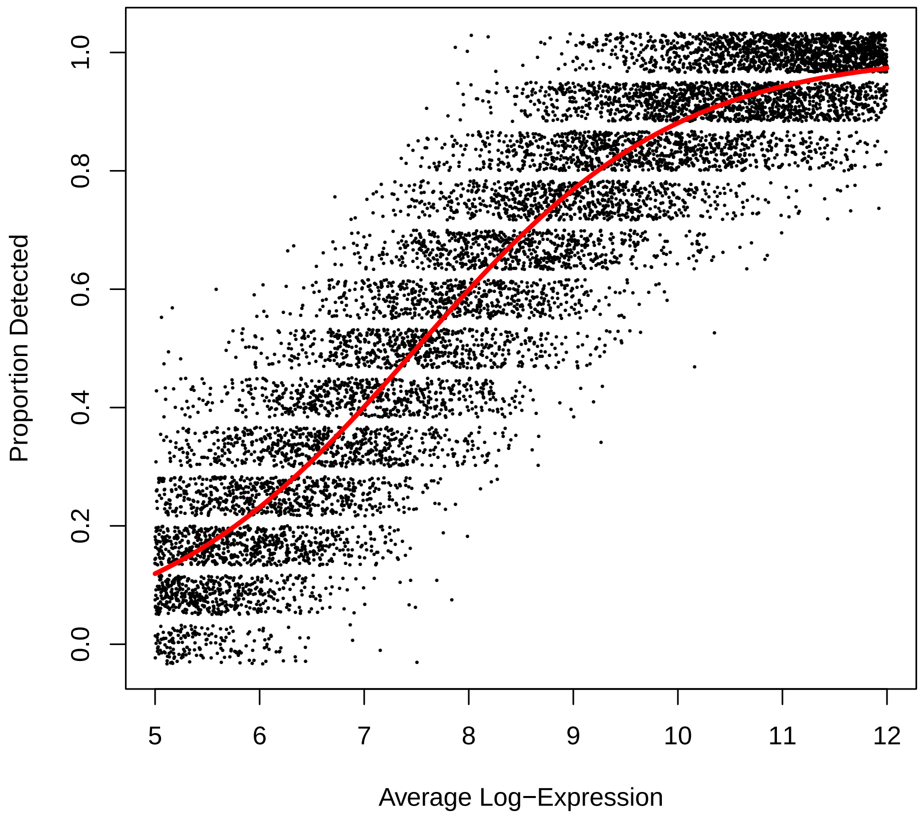

Figure S8: Detection probability curve for simulated data.

3 Supplementary Tables

Table S1: Percentage of total reduced deviance explained by the logit-linear and non-linear regression splines fitted on the protein group-level data shown in Figure S1. The rows of the table show the percentage of the total deviance explained by the the logit-linear curve and the additional deviance explained by logit splines with 3 or 5 degrees of freedom.

	Deviance Explained (%)			
**Source**	**A**	**B**	**C**	**D**
Linear	97.1385	97.0106	97.4772	97.9619
Nonlinear 2—3	2.0038	2.1600	1.7366	1.9275
Nonlinear 4—5	0.8578	0.8295	0.7862	0.1106

Table S2: Parameter estimates for detection probability curves fitted on the protein group level for Datasets A–D visualized in Figure S5.

		Detection probability curve	
**Dataset**	**Fitted on**	β_0	β_1
A	Observed	-1.8880	0.8960
	Underlying	-1.7533	0.8731
B	Observed	-13.8707	1.2662
	Underlying	-12.9501	1.1976
C	Observed	-11.9163	0.5480
	Underlying	-11.9005	0.5476
D	Observed	-11.9030	0.7933
	Underlying	-10.8627	0.7765

Table S3: Parameter estimates for detection probability curves fitted on Datasets E and F in Figures S6f–g and S7f–g.

		Detection probability curve		
**Dataset**	**Level of quantification**	**Fitted on**	β_0	β_1
E	Precursor	Observed	-7.8223	0.5197
		Underlying	-7.7910	0.5184
	Protein group	Observed	-7.1560	0.5761
		Underlying	-7.0890	0.5729
F	Precursor	Observed	-12.5691	0.6063
		Underlying	-12.4444	0.6015
	Protein group	Observed	-17.2926	0.8356
		Underlying	-17.1109	0.8295

Table S4: Performance for differential expression. The table gives the true positive rate (TPR), false discovery rate (FDR) and reverse discovery rate (RDR) for various methods for handling missing values when assessing differential expression between two groups. Reverse discoveries refer to features correctly identified as differentially expressed but in the wrong direction. limma was used for all analyses except “DPC”. Numbers are percentages averaged over 10 simulated datasets using FDR < 0.05. “Complete” gives results from full data without missing values. “Missing” gives results with missing values left in the data. “DPC” gives results from likelihood ratio tests using the DPC. “v1”, “v2” and “v2nmar” are imputation methods implemented in the msImpute package. “Perseus” is the imputation strategy used by Perseus software. The remaining are imputation methods implemented by the MSnbase and MsCoreUtils packages.

	**Metric**		
**Method**	**TPR(%)**	**FDR(%)**	**RDR(%)**
Complete	99.85	4.46	0.000
Missing	75.08	3.95	0.000
DPC	76.72	5.00	0.000
v1	63.27	3.33	0.000
v2	75.34	15.06	0.022
v2mnar	64.76	8.22	0.000
bpca	85.59	51.93	0.017
MLE	79.68	41.94	0.926
RF	61.85	4.98	0.000
knn	44.96	4.05	0.534
MinDet	12.60	9.29	0.000
MinProb	8.58	7.94	0.000
QRILC	0.00	100.00	0.000
Perseus	8.91	4.81	0.000

References

[1] Demichev,V., Messner,C.B., Vernardis,S.I., Lilley,K.S. and Ralser,M. (2020) DIA-NN: neural networks and interference correction enable deep proteome coverage in high throughput. *Nature methods*, **17**, 41–44.

[2] Cox,J., Hein,M.Y., Luber,C.A., Paron,I., Nagaraj,N. and Mann,M. (2014) Accurate proteome-wide label-free quantification by delayed normalization and maximal peptide ratio extraction, termed MaxLFQ. *Molecular & cellular proteomics*, **13**, 2513–2526.

[3] Cox,J. and Mann,M. (2008) MaxQuant enables high peptide identification rates, individualized ppb-range mass accuracies and proteome-wide protein quantification. *Nature biotechnology*, **26**, 1367–1372.

[4] Li,M., Parker,B.L., Pearson,E., Hunter,B., Cao,J., Koay,Y.C., Guneratne,O., James,D.E., Yang,J., Lal,S. *et al.* (2020) Core functional nodes and sex-specific pathways in human ischaemic and dilated cardiomyopathy. *Nature communications*, **11**, 1–12.

[5] Perez-Riverol,Y., Bai,J., Bandla,C., García-Seisdedos,D., Hewapathirana,S., Kamatchinathan,S., Kundu,D.J., Prakash,A., Frericks-Zipper,A., Eisenacher,M. *et al.* (2022) The PRIDE database resources in 2022: a hub for mass spectrometry-based proteomics evidences. *Nucleic acids research*, **50**, D543–D552.

[6] Giai Gianetto,Q., Combes,F., Ramus,C., Bruley,C., Couté,Y. and Burger,T. (2016) Calibration plot for proteomics: A graphical tool to visually check the assumptions underlying FDR control in quantitative experiments. *Proteomics*, **16**, 29–32.
